# Worse Neurological State During Acute Ischemic Stroke is Associated with a Decrease in Serum Albumin Levels

**DOI:** 10.1007/s12031-015-0705-4

**Published:** 2016-01-12

**Authors:** Joanna Bielewicz, Jacek Kurzepa, Elżbieta Czekajska-Chehab, Piotr Kamieniak, Beata Daniluk, Halina Bartosik-Psujek, Konrad Rejdak

**Affiliations:** Department of Neurology, Medical University of Lublin, Jaczewskiego 8, 20-954 Lublin, Poland; Department of Medical Chemistry, Medical University of Lublin, Chodźki 4a, 20-093 Lublin, Poland; 1st Department of Radiology, Medical University of Lublin, Jaczewskiego 8, 20-954 Lublin, Poland; Department of Neurosurgery, Medical University of Lublin, Jaczewskiego 8, 20-954 Lublin, Poland; Institute of Psychology, Marie Curie-Skłodowska University in Lublin, Plac Litewski 5, 20-080 Lublin, Poland; Department of Neurology, University of Rzeszow, Lwowska 60, 35-301 Rzeszow, Poland

**Keywords:** Ischemic stroke, Albumin, S100BB protein, Outcome

## Abstract

High serum albumin levels during ischemic stroke (IS) decrease the risk of a poor outcome. This study aimed to determine whether serum albumin levels within the first days after IS correlate with radiological and biochemical markers of brain tissue damage. Fifty-six IS patients were enrolled into the study. Neurological examinations were based on the National Institute of Health Stroke Scale. Serum albumin levels and S100BB were evaluated using commercially available ELISA kits. The albumin decrease index (ADI) was calculated as the difference between serum albumin levels measured on days 1 and 10 of IS. All parameters were estimated on the 1st, 3rd, 5th, and 10th days of IS, and the volume of ischemic focus was measured on the 10th day. Mean serum albumin levels were decreased during acute IS. There were correlations between the ADI and mean S100BB serum levels (*r* = 0.36, *p* < 0.05), the volume of ischemic focus (*r* = 0.39, *p* < 0.05), and the patients’ neurological state when measured on day 10 of IS (*r* = 0.59, *p* < 0.001). A decrease in serum albumin levels during the acute phase of IS corresponds to a worse neurological state as a result of a large ischemic focus with intense catabolic processes.

## Introduction

Previous studies have highlighted the favorable relationship of serum albumin levels on the outcome of patients experiencing ischemic stroke (IS) (Babu et al. 2013; Baltanas et al. 2009; Belayev et al. 2002). Small ischemic lesions are independently correlated with high serum albumin levels (Boselli et al. 2012). Moreover, a low serum albumin level is one of the predictive factors for a first-ever non-embolic stroke in older individuals (Buttner et al. 1997). Some experimental studies have suggested a neuroprotective effect of albumin either by reducing brain edema (Chen et al. 2013) or by its antioxidative or antiapoptotic properties (Ciuffetti et al. 1988). Adequate levels of serum albumin lead to an improvement in microcirculatory flow, viscosity of plasma, and oxygen transport capacity (Defazio et al. 2012). Serum albumin levels also have a beneficial influence on the immune system (Dziedzic et al. 2012). Local, low-dose, cold albumin infusion into the ischemic area enhances neuroprotection (Dziedzic et al. 2006). Albumin treatment exerts a significant therapeutic effect after ischemia by augmenting collateral perfusion (Dziedzic et al. 2004).

This study aimed to determine whether baseline serum albumin levels or a change in levels within the first few days after IS correlates with the patient’s neurological state and with radiological or biochemical markers of brain tissue damage.

## Subjects and Methods

Fifty-six patients, who had acute IS confirmed in a computed tomography (CT) scan and were admitted to the Stroke Unit at the Department of Neurology, Medical University of Lublin, were prospectively enrolled into the study. The main qualification criterion was the time when the patient was seen without focal neurological symptoms that did not exceed 12 h. Other neurological disorders that could influence the S100BB serum level excluded patients from the study. Written informed consent was obtained from each patient (or from family members when necessary). The local ethics committee accepted the protocol of the study.

The characteristics of the study and control groups are shown in Table 1. Neurological examinations were performed on days 1, 3, 5, and 10 from admission to the hospital based on the National Institute of Health Stroke Scale (NIHSS).

**Table 1:** Patients’ characteristics

Mean age (range)	72.8 (48–93) years
Sex	Male *n* = 24, female *n* = 32
OCSP classification	TACI *n* = 6
	PACI *n* = 31
	LACI *n* = 10
	POCI *n* = 9
Thrombolytic therapy	*n* = 3

*OCSP* The Oxfordshire Community Stroke Project classification, *TACI* total anterior circulation infarct, *PACI* partial anterior circulation infarct, *LACI* lacunar infarct, *POCI* posterior circulation infarct

CT scans were performed without contrast scanning, using 64-row multidetector CT (LightSpeed VCT with Advantage Window 4.3 workstation). The first CT scan was performed upon admission to hospital and a second scan was performed on 10 days after the stroke for estimation of ischemic focus volume (IF). CT scans were 2.5 mm in the posterior fossa and 5 mm in other areas of the brain. IF was measured with the planimetric method, using an additional workstation for measuring IF in the three-dimensional option.

Five milliliters of venous blood samples were obtained on 1, 3, 5 and 10 days of IS for the measurement of albumin and S100BB protein levels. Patients were fasted for at least 8 h before blood collection. After the clot forming the blood was centrifuged and serum was transferred into the temperature −70 for further analysis.

Serum albumin levels were evaluated with usage of commercially available enzyme-linked immunosorbent assay (ELISA) kit (Cell Biolabs, Inc., San Diego, CA) according to the manufacturer’s instructions. Before the analysis, the sera were diluted 1:10^6^ with phosphate-buffered saline (PBS) to adjust the albumin concentration to the range of sensitivity of the applied method. The albumin decrease index (ADI) was calculated as the difference in baseline serum albumin levels measured on days 1 and 10 of stroke.

S100BB serum levels were measured as the biochemical marker of traumatic and vascular brain damage (Laribi et al. 2014; Brea et al. 2009; Ishibashi and Funakoshi 2008) and as an indicator of blood-brain barrier (BBB) integrity (Koh and Lee 2014). Commercially available ELISA kit (CanAg Diagnostics AB, Gothenburg, Sweden) was applied to S100BB analysis according to the manufacturer’s instructions. The detection limit of the S100BB protein was 10 pg/ml. All values below the detection limit were rendered as zero and were not applied in calculations.

Spearman’s rank correlation was applied to calculate the relationship between biochemical parameters and CT findings or NIHSS values. Significant values were considered when *p* was <0.05. Statistical analysis was performed with the use of the computer-assisted software, GraphPad InStat v. 3.06. (San Diego, USA).

## Results

Baseline serum albumin levels were not correlated with mean S100BB serum levels (*r* = 0.16, *p* > 0.05) or the patients’ neurological state (correlation with NIHSS on day 10 of stroke: *r* = 0.12, *p* > 0.05). However, baseline serum albumin levels predicted IF (*r* = 0.35, *p* < 0.05). ADI was significantly correlated with mean S100BB serum levels (*r* = 0.36, *p* < 0.05), IF (*r* = 0.39, *p* < 0.05), and the patients’ neurological state (NIHSS score) on day 10 of stroke (*r* = 0.59, *p* < 0.001). The ADI was not significantly different between different subgroups of the Oxfordshire Community Stroke Project (OCSP) classification (*p* > 0.05, Kruskal-Wallis test). The values of serum albumin and S100BB protein levels are shown in Table 2.

**Table 2:** Changes in albumin levels, S100BB serum levels, and NIHSS scores during the acute phase of ischemic stroke

	Day 1	Day 3	Day 5	Day 10	*p*
Albumin (g/dl)	4.04 (0.42)	3.80 (0.44)	3.80 (0.43)	3.72 (0.49)	0.006^*^
S100BB (pg/ml)	51.02 [30.15; 77.17]	57.95 [27.49; 109.06]	52.96 [30.80; 92.56]	36.00 [25.56; 59.24]	0.060^†^
NIHSS	13 [7; 18]	10.5 [5; 16]	9 [4.25; 15]	9 [3.25; 14]	0.045^†^
Volume of IF (ml)				17.00 [2.09; 67.48]	
ADI				0.32 [−0.10; 0.60]	

The volume of ischemic focus (IF) and the albumin decrease index (ADI) were estimated on day 10 of stroke. Values are expressed as mean (SD) or median (1st quartile; 3rd quartile)

*ANOVA

^†^Kruskal-Wallis test

## Discussion

This study showed a gradual decrease in serum albumin levels in patients during acute IS. We observed interesting relationships between ADI and IF mean S100BB levels, and the NIHSS score. A greater IF and higher S100BB serum levels, which partially reflect the size of ischemic focus as well as the state of the BBB, were observed in patients with a greater ADI. Some experimental and histological studies have shown extravasation of plasma proteins into the ischemic focus in angiogenesis during clinical recovery (Kapural et al. 2002). In addition, ADI can reflect an increase in the metabolic rate within the infarcted area or a decrease in albumin synthesis during the acute phase of IS. Release of hormones, such as glucagon, catecholamines, and corticosteroids, results in intensification of numerous catabolic pathways, including proteolysis. These processes are connected with the degradation of serum albumin, which then leads to hypoalbuminemia. Low serum albumin levels in patients with IS are associated with higher serum cortisol levels (Kisialiou et al. 2012). We suggest that ADI during stroke can indirectly reflect the intensity of the response within the ischemic focus. A prolonged and intense catabolic state might also lead to insufficiency of immune responses that make patients prone to numerous complications, such as infections (Liu 1988) or loss of weight (Milionis et al. 2005). This may play a part in a worse outcome after stroke, which was confirmed in our study. Oral supplementation of essential amino acids may reduce the occurrence of nosocomial infection in patients with brain injury (Wang et al. 2013).

We conclude that worsening of the neurological state corresponds to a decrease in serum albumin levels as a result of a large ischemic focus with intense catabolic processes during acute IS.

## Abbreviations

ADI: Albumin decrease index (difference in serum albumin levels measured on days 1 and 10 after ischemic stroke)
BBB: Blood-brain barrier
IF: Ischemic focus volume
IS: Ischemic stroke
NIHSS: National Institute of Health Stroke Scale
